# Genotyping of dengue virus from infected tissue samples embedded in paraffin

**DOI:** 10.1186/s12985-023-02072-5

**Published:** 2023-05-25

**Authors:** Jorge Alonso Rivera, Aura Caterine Rengifo, Alicia Rosales-Munar, Taylor H. Díaz-Herrera, José Usme Ciro, Edgar Parra, Diego A. Alvarez-Díaz, Katherine Laiton-Donato, María Leonor Caldas

**Affiliations:** 1 Dirección de investigación en Salud Pública, Grupo de Morfología Celular, Instituto Nacional de Salud, Avenue 26 No. 51-20 – Zone 6 CAN, Bogotá, Colombia; 2 Dirección de Redes en Salud Pública, Grupo de Patología, Instituto Nacional de Salud, Bogotá, Colombia; 3 Dirección de investigación en Salud Pública, Grupo de Genómica de Microorganismos Emergentes, Instituto Nacional de Salud, Bogotá, Colombia; 4 CIST-Centro de Investigaciones en Salud Para el Trópico, Facultad de Medicina, Universidad Cooperativa de Colombia, Santa Marta, 47003 Colombia

**Keywords:** Dengue virus, Genotype, Paraffin-embedded tissues, Colombia

## Abstract

Dengue has become one of the vector-borne diseases that affect humans worldwide. In Latin American countries, Colombia is historically one of the most affected by epidemics of this flavivirus. The underreporting of signs and symptoms of probable cases of dengue, the lack of characterization of the serotypes of the infection, and the few detailed studies of postmortem necropsies of patients are among other conditions that have delayed progress in the knowledge of the pathogenesis of the disease. This study presents the results of fragment sequencing assays on paraffin-embedded tissue samples from fatal DENV cases during the 2010 epidemic in Colombia. We found that the predominant serotype was DENV-2, with the Asian/American genotype of lineages 1 and 2. This work is one of the few reports of the circulating genotypes of dengue during the 2010 epidemic in Colombia, one of the most lethal dates in the country's history.

**Supplementary Information:**

The online version contains supplementary material available at 10.1186/s12985-023-02072-5.

## Introduction

Dengue virus (DENV) occurs in more than 125 tropical and subtropical countries worldwide, with an estimated 390 million infections and 90 million symptomatic cases annually; Its risk of transmission poses one of the most significant health challenges to the public [1–3]. Any of the four serotypes (DENV-1 to DENV-4) can be responsible for the disease and manifest an asymptomatic form or a severe form known as severe dengue [4]. Severe dengue form could be related to secondary exposure to heterologous serotypes, host-specific genetic background, or the presence of a specific viral serotype or genotype highly infecting [5, 6]. Each of the serotypes responsible for the infection is subdivided into several genotypes, defined as a group of DENV isolates that usually do not present more than 7.2% divergence in their nucleotide sequence [7, 8]. Five genotypes have been defined for DENV-1, six for DENV-2, five for DENV-3, and four for DENV-4. For example, DENV-2 can be divided into six genotypes (Asian I, Asian II, American, Asian/American, Wild, and Cosmopolitan) [8–10]. Different genotypes of the same serotype may vary in their ability to infect host cells [11] and tcause severe forms of the disease. Phylogenetic analysis of envelope gene (E gene) sequences has shown that each genotype can be subdivided into several lineages that in turn, may have a greater involvement in the development of the disease [10, 12, 13]. Individuals with severe dengue who need immediate medical attention typically have substantial plasma loss, resulting in hypovolemic shock or fluid buildup in the lungs with respiratory difficulty. These patients may also exhibit multiorgan failure, which includes, among other things, cardiomyopathy, liver damage, and renal failure [14]. Routine histopathology, detection, and localization of viral genomes and antigens studies; have shown that organs such as the liver, lung, and kidney are the main organs affected by viral infection [14–17].

The fixation of tissues in formalin and their inclusion in paraffin blocks is the technique of choice for the histopathological study because it preserves the morphology and integrity of the proteins in the tissue. In addition, it is very useful to preserve these samples for long periods. However, the nucleic acids extracted from this type of sample are generally of low quality due to their processing, presenting modifications such as fragmentation, crosslinking, and formation of basic sites that lead to localized denaturation of DNA and chain breaks. Another phenomenon in this type of tissue is deamination, which leads to mutation such as C > T; these modifications are important for sequencing studies [18–20].

Colombia experienced one of the worst DENV epidemics in its history in 2010, with roughly 151,983 cases and 217 deaths, equivalent to a case fatality rate of 2.28%. In subsequent years, the case fatality rate increased to 6.2% in 2014, but the number of infected registered not exceed the number of cases reported in 2010 [21]. Although there are reports of infectious serotypes in fatal cases, there are few reports on the genotypes circulating during this epidemic. This study shows the results of DENV-2 genotyping by amplification, sequencing, and assembly of small fragments of the E gene from formalin-fixed and paraffin-embedded samples from fatal cases of DENV in 2010. We present small fragment sequencing as a good alternative for the molecular study of this type of sample.

## Materials and methods

### Sample handling and ethical considerations

The selected samples were obtained from patients who died from severe dengue during the epidemiological outbreak of 2010 in Colombia. The Virology and Pathology laboratories of the National Institute of Health-Bogotá Colombia (INS) confirmed 97 cases of the 217 deaths from DENV reported in 2010 [21]. For the sequencing experiments, tissues from the 97 cases that showed a Ct of 35 cycles or less in rt-qPCR for DENV-2 assays were chosen (liver, spleen, kidney, heart, lung, and brain samples embedding in paraffin) [22]. A total of 22 tissue samples met this criterion. The 22 selected tissue samples underwent viral antigen localization assays and routine histology analysis according to protocols previously standardized [23]. Antigen localization was performed on 4-µm thick sections with the anti-DENV antibody VS0090 (Immune mouse ascitic fluid—Donated by the Center for Disease Control CDC, Atlanta, Georgia) at 1:800 dilution.

The protocols used in this study were approved by the ethics and research methodologies committee of the INS, project CTIN 24 of 2015. This study followed the basic ethical principles promulgated in the Declaration of Helsinki adopted by the 18th World Medical Assembly, Helsinki. Finland, June 1964, where it is indicated that specific consent is not required for the use of the samples [24]. The Colombian regulations for health research resolution 8430 of 1963 chapter VI [25] also took into account ethical guideline 11 for research related to health with humans of the Council for International Organizations of Medical Sciences (CIOMS) [26]. In addition, law 2323 of 2006 was considered, which indicates that the INS, as a reference laboratory and health authority of the national network of laboratories, may use biological material for public health research purposes without informed consent, provided that the anonymous disclosure of the results is maintained [27].

### Extraction of RNA from tissues embedded in paraffin

Total RNA was extracted from each tissue block embedded in paraffin up to two blocks per organ to perform the viral amplicon amplification and sequencing. For RNA extraction, 10 µm thick sections were performed on the tissue block until 100 µm per block was obtained. Subsequently, the sections were placed in a 1.5 mL tube with 1 mL of xylol (Merck, USA), centrifuged at 15,000 rpm for 1 min, vigorously stirred, and centrifugation was repeated for 1 min. Subsequently, 1 mL of molecular grade ethanol (Merck, USA) was added and centrifuged at 15,000 rpm for 1 min. Then, the ethanol was carefully removed and allowed to evaporate. Next, 500 µL of lysis buffer with proteinase K (10 mM Tris–HCl, 2 mM EDTA, 1% SDS; 0.2 µg/µL proteinase K) was added and incubated at 56 °C for 24 h [28]. Subsequently, the mixture was incubated at 80 °C for 15 min and left at − 20 °C for 3 min. From this step, RNA extraction was continued using the RNeasy Mini-kit (Qiagen,Germany) following the protocol recommended by the manufacturer. The RNA obtained was stored at − 80 °C for later use.

### Identification of DENV serotypes

Conventional RT‒PCR and real-time RT‒PCR assays previously reported were performed to establish the methodology to be followed [29, 30]; however, only the four-probe Taqman system described by Jhonson et al. was able to amplify viral RNA in the samples tested [21]. Reverse transcription RT and PCR were performed in a single step using the SuperScript III Platinum One-Step qRT‒PCR Kit (Invitrogen USA).

The reaction conditions were as follows: reaction buffer with magnesium sulfate (MgSO_4_) 3 mM and 0.2 mM of each dNTP, 1 μM primers for serotypes 1 and 3 and 0.5 μM for serotypes 2 and 4; reference fluorophore ROX ™ 0.083 μM and RNase inhibitor (RNaseOut™) 0.5 U/μL (Invitrogen, USA); the volume of total RNA extract was 3.7 μL; and the reaction was brought to a final volume of 15 μL. All samples were evaluated in duplicate.

The thermal profile for cDNA synthesis and amplification was as follows: cDNA synthesis at 50 °C for 15 min, inactivation of the reverse transcriptase enzyme, and activation of DNA polymerase at 95 °C for 2 min, followed by 40 denaturation cycles at 95 °C for 15 s and annealing and polymerization at 60 °C for 30 s. These same samples were tested for DENV-2 negative strands to assess viral replication.

### Amplification of envelope gene fragments (E) for DENV-2

To obtain amplicons for sequencing of DENV E gene fragments, samples with a Ct less than or equal to 35 were selected based on the recommendations of Johnson et al*.* for the DENV detection assays by real-time PCR [22].

From the total RNA obtained from the paraffin-embedded tissues, conventional PCR amplification of DENV-2 E gene fragments was performed using the primers designed by Santiago et al. Performing combinations of primers to obtain smaller fragments is shown in Table 1 [31]. The SuperScript III One-Step RT‒PCR system with Platinum Taq (Invitrogen, USA) was used following the manufacturer’s recommendations.

**Table 1:** Primers for the amplification and sequencing of DENV-2 envelope gene fragments

Primer	MIX primer	Sequence	Tm (°C)	Position in gene E	Product size (pb)
D2SEQ1-F	1	TYGCTCCTTCAATGACAATGCG	59.3	920–1341	422
D2SEQ8-R		CAARTTTTCTGGTTGCACGACT	57.3		
D2SEQ2-F	2	ACATGCAAAAAGAACATGGAAGGA	57.5	1294–1732	439
D2SEQ7-R		CTGTGAGTGCCGTGTGCATG	59.2		
D2SEQ3-F	3	AATCCCCAYGCVAAGAAACAGGAT	62.5	1660–2074	415
D2SEQ6-R		TGATGATGTAGCTGTCTCCGAATG	58.3		
D2SEQ4-F	4	CCATTCGGRGACAGCTACATCAT	57.9	2050–2444	395
D2SEQ5-R		GAC CTC ACA GCA ACA CCA CTA	58.4		

The primers were based on the sequences reported by Santiago et al. [27]

The synthesis of cDNA and the amplification of the fragments were obtained with the following thermal profile: 55 °C for 20 min, 94 °C for 2 min, 40 cycles (94 °C for 15 s, 54 °C for 30 s, 65 °C for 30 s), 65 °C for 5 min and 4 °C.

### Purification of PCR products

The purification of highly specific PCR fragments lacking nonspecific products and primer dimers was performed with a commercial *QIAquick PCR purification kit* QIAquick PCR purification kit (Qiagen, Germany) following the manufacturer’s instructions. Thirty microliters of the purified product were obtained per sample and stored at − 80 °C for subsequent sequencing.

### Direct Sanger sequencing and sequence editing

The envelope gene fragments were sequenced using Sanger sequencing techniques through the company Macrogen Inc. (Seoul, Korea). The samples were prepared according to the instructions of the manufacturer Macrogen Inc. The sequences obtained were edited and assembled using the software Geneious®2016.9.1.8 (https://www.geneious.com).

### Phylogenetic analysis

The sequences of gen E-DENV-2 fragment from the fatal cases obtained in the present research were aligned using the Muscle algorithm with default settings. The alignment employed eight nucleotide sequences from the fatal cases, twenty-four nucleotide sequences reported in Colombia, and 55 reference sequences from DENV-2 genotypes. The nucleotide sequences reported in Colombia and the reference sequences of the DENV-2 genotypes are available in the GenBank database [32].

We used Mega X software to build the phylogenetic tree from the obtained alignment of ~ 366 nucleotides, corresponding to the partial gene E. The phylogenetic analysis was done through the maximum likelihood method, with 500 bootstrap replicates [33].

## Results and discussion

### Histological findings

**Fig. 1:**
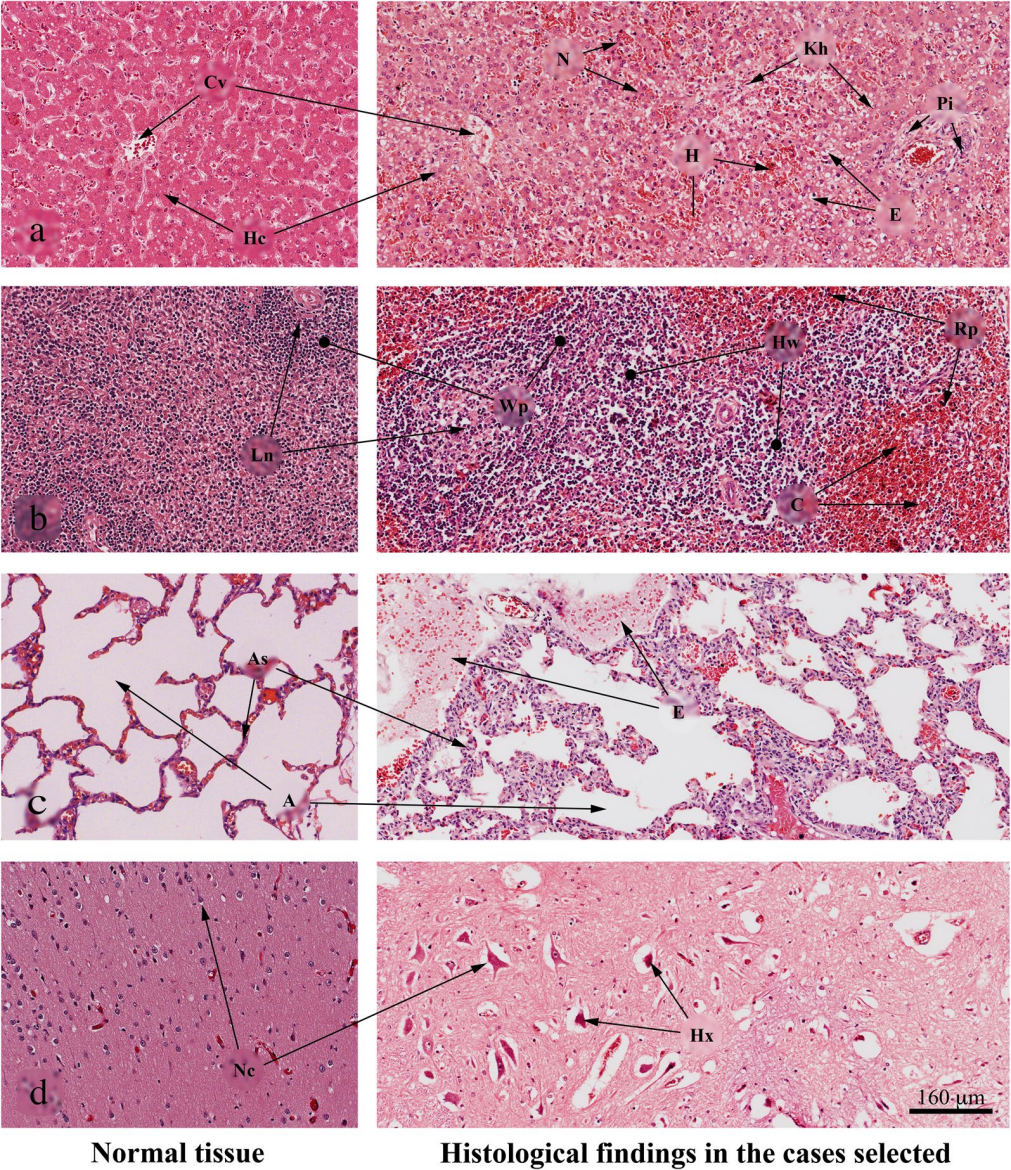
Morphological changes observed in tissues from selected cases. **a** Hepatic tissue. Note the loss of the radial arrangement of the hepatic plaques with pale eosinophilic staining and pyknotic or absent nuclei; Cv-Central vein, Hc-Hepatic cells, N-necrosis, H-Hemorrhage, Kh-Kupffer cells hyperplasia, E-Esteaosis, Pi-Portal tract infiltrate. **b** Splenic tissue. The image mainly shows an enlargement of the white pulp and congestion of the venous sinuses; Ln-Lymphoid node, Wp-White pulp, Hw-Hyperplasia white pulp, C-Congestion, Rp-Reactive plasmacytosis. **c** Pulmonary tissue. Note the septum thickening and serous fluid in the alveolus (Edema).; As-Alveolar septum, A-Alveoli, E, Edema and **d** Cerebral tissue. The image shows a decrease in neuronal size due to retraction of the cytoplasm, with pyknosis and hyperchromasia of the nucleus; Nc-Neuronal cell, Hx-Hipoxia, Hematoxylin and Eosin coloration (H&E)

A total of 22 samples from all selected tissues showed positive qRT-PCR for DENV-2 with a Ct value according to the criteria mentioned previously in the methodology. These tissues were processed to obtain the DENV E gene fragments, but some of them did not show immunostaining for the DENV-2 antigen (see Additional file 1: Table S1). These organs also showed morphological changes associated with the viral infection. One of the most severely affected organs was the liver, which mainly presented inflammatory cell infiltration in the portal tract in 70.1%, Kupffer cell hyperplasia in 82.8%, necrosis in 78.6%, and macro and microvesicular steatosis in 56.3% (Fig. 1). These findings agree with those of other studies showing that the DENV virus causes liver damage, sometimes leading to irreversible hepatocellular diseases [34–36]. The detection of the virus, the observed morphological alterations, the localization of viral antigens, and the detection of RNA of replication (negative strand) in the liver (Additional file 1: Table S1) suggest that DENV has a tropism for the organs of the monocyte-macrophage system as has been reported in other studies in those showing this tropism for organs such as bone marrow, spleen, liver, and lymph nodes [34, 37]. In addition to being positive in real-time PCR for DENV, many of the organs evaluated presented viral antigens and negative strands, all of which are evidence of marked viral tropism towards different target organs (see Additional file 1: Table S1). These findings are of great interest because most of the fragments that could amplify during the conventional PCR assays were obtained from liver samples, which could be associated with a higher viral load and therefore higher tropism [38].

### Successful amplification of DENV-2 E gene fragments

**Fig. 2:**
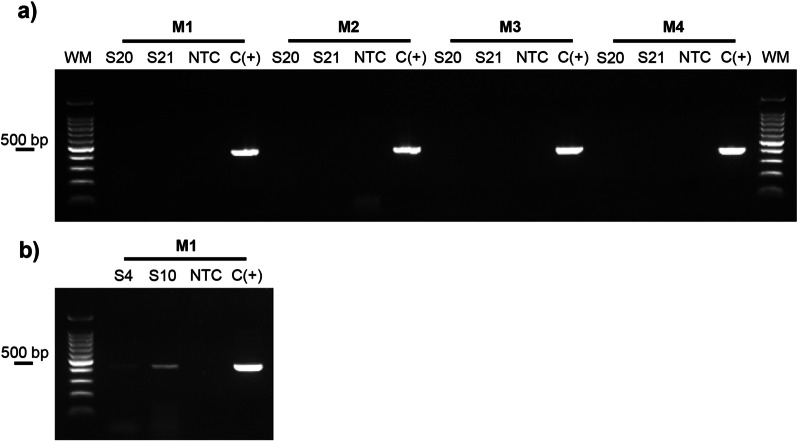
Amplification products of the pairs of primers used to obtain fragments of the DENV-2 E gene. **a** Expected amplification products, **b** amplification products obtained for some samples (S20, S21, S4, S10). **S** letter corresponds to the coding assigned to the samples of 2% agarose gels stained with SYBR safe, as shown in Additional file 1: Table S1. M1–M4 abbreviations of the primer mixture are described in Table 1

Twenty-two samples positive for DENV-2 were selected to meet the criterion of a Ct less than 35. The quality of the total RNA was estimated by Nanodrop through the ratios between the absorbance 260/230 and 260/280 nm as purity indicators. For each sample, values at 2.0 were obtained (see Additional file 1: Table S1), which suggests that the deparaffinization process described in this study contributes to obtaining total RNA useful for molecular assays [39]. The samples that presented a Ct less than or equal to 35 corresponded to those infected with serotype DENV-2 (Table 2). In all the tissue samples evaluated, the viral genome was detected by real-time RT‒PCR; however, when amplification of the envelope gene fragments was performed through conventional PCR, it was not possible to find amplification products of greater size in all cases, as shown in Fig. 2 and Additional file 2: Fig. S1. This result coincides with reports of fragmentation of nucleic acids of this type of sample, which is reflected in the difficulty of obtaining larger fragments, as occurs in the assays for conventional PCR [40]. Figure 2 shows the expected amplification products and those observed in some samples for the envelope gene of DENV-2; The expected fragment size is, on average, 409 bp according to the primer combinations listed in Table 1, and the details on these samples are presented in Additional file 1: Table S1; here the fragment sizes obtained and the associated GenBank code are shown.

**Table 2:** Genotyping of DENV-2 E gene in tissue samples embedded in paraffin

Case no	Pathology laboratory code	Ct	Sample ID	Mix primer^§^	Product size (pb)	Genotype	Lineage
S1		27.1	Liver	2	392	Asian/American	2
S2	59360	30.9	Lung		–		
S3	59404	28.8	Liver		–		1
S4		27.1	Spleen	1	366		
S5		26.2	Liver		–		2
S6	59439	34.8	Lung		–		
S7		28.6	Spleen	1	427		
S8		33.2	Heart		–		
S9	59461	24.6	Liver	Indeterminate			
S10	59479	28.8	Liver	1	426	Asian/American	1
				4	396		
S11		26.9	Liver-1	Indeterminate			
S12	59484		Liver-2				
S13	59569	28.8	Liver	3	419	Asian/American	2
S14		28.9	Spleen		–		
S15		32.4	Lung		–		
S16		32.3	Heart		–		
S17		30.4	Liver-1	4	369		1
S18	59616		Liver-2		–		
S19	59702	28.7	Liver	Indeterminate			
S20		30.1	Lung				
S21	59888	28.5	Liver	4	396	Asian/American	1
S22		30.3	Lung		–		

*The minus symbol corresponds to samples in which no amplification was obtained

^§^Mix of primers indicated in the Table 1

### Phylogenetic analysis

We used 79 sequences representatives of each genotype and lineage circulating during the 1998–2016 period to calculate the intra-lineage and inter-lineage distances of the phylogenetic reconstruction. The maximum likelihood method with the stochastic Tamura-Nei nucleotide substitution model were used [33]. This model has been previously used for other phylogenetic analysis using maximum likelihood methods [41].

**Fig. 3:**
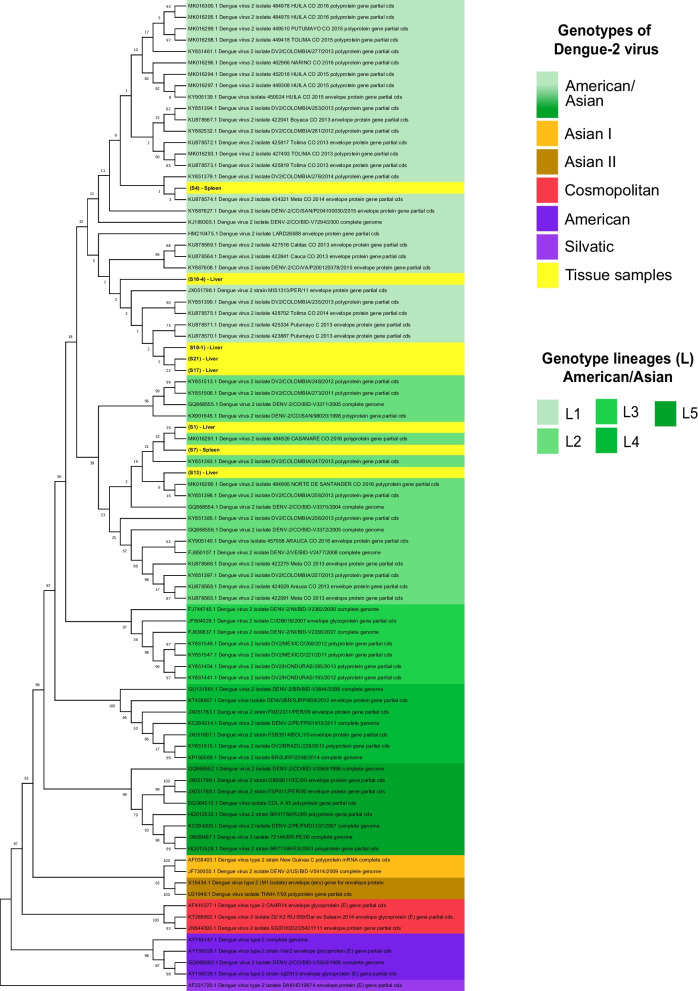
Phylogenetic analysis of DENV-2 based on reference sequences of each genotype and lineage

According to the phylogenetic analysis (Fig. 3), the samples of this study are part of the Asian/American genotype. This genotype groups some ancestral strains of Southeast Asia and those of the Caribbean region and Latin American countries [9, 42]. The Asian/American genotype is associated with a significant increase in DHF in the region [40] with a displacement of less virulent strains such as the American genotype, in the Americas that have caused large epidemics with greater pathogenicity [11, 43, 44]. Although the analyzed samples belong to the same genotype, they were defined in two lineages (lineage 1 and lineage 2), and it has been shown that different lineages within the same genotype or different genotypes of the same serotype can be key in the development of severe disease [42, 45].

Williams et al. observed higher virus load in cells infected with genotype II of DENV-2 after comparing the kinetics of viral replication in C6/36 and Huh-7 cells [46]. Hence, the level of damage in severe dengue cases and the different organs assessed in our report could be linked to infection capability of this genotype.

In 2010, Brazil had a sizeable epidemiological outbreak following the introduction of DENV-2 belonging to the Asian/American genotype [47]. In the same year, Colombia suffered one of the largest epidemics of DENV, whose fatal cases presented multiorgan alterations consistent with what was presented here. Since this was a retrospective study, it was unfortunate that the whole clinical history of the subjects examined was not accessible. Table S2 summarizes the scant data collected. Here, we only can infer that all patients had the typical clinical symptoms of severe dengue, including myalgia, arthralgia, abdominal pain, and hemorrhages. In most cases, thrombocytopenia and high hematocrit levels were found, which might be signs of vascular leakage. Alterations in liver transaminases were noted in three cases, although the data evaluated for this marker or others were unavailable for the previously described reasons.

In our study, it is possible to infer that the severity of the cases and the fatal outcome may be associated with the circulation of the Asian/American genotype of DENV-2 and its capacity to invade, replicate, and affect the integrity of the host. Several studies reinforced this hypothesis that DENV-2 genotypes have a higher rate of viral replication than other DENV serotypes, contributing to an aggravating variable among other factors that determine a fatal outcome [48].

Complete sequences of the E gene in 48 DENV strains have demonstrated antigenic evolution. The differences in amino acids or mutations that this gene presents in DENV have been correlated with its potential for infection and its severity [49]. On the other hand, studies conducted in Colombia have documented results similar to those reported in this study. The presence of the DENV-2 Asian/American lineage has been reported in Santander, Antioquia, Guaviare, and Valle del Cauca [32]. Additionally, the evolutionary history of this lineage in Colombia presents similarities and phylogenetic relationships with other areas of South and Central America (Lesser Antilles, Venezuela, and Costa Rica) [32, 45]. The viral spread of the Asian/American lineage in Colombia is mainly attributed to the migratory proximity to Venezuela [50]. In complete sequences of the E gene in DENV-2, three variants (Ia, Ib and II) have been described, with a differential genetic degree to the other serotypes, so sequencing the envelope gene could be an important molecular marker for epidemiological surveillance laboratories [51].

## Conclusions

It was determined that the DENV-2 Asian/American genotype was present in the fatal cases of the samples of the 2010 dengue outbreak in Colombia, consistent with previous reports of this lineage and its circulation in the country [45, 52]. These studies have identified viral types from isolates in C6/36 cells from sera of DENV-infected patients. However, our study is the first report to present viral types in different organ types about morphological changes associated with viral infection during this large viral outbreak. On the other hand, our manuscript highlights the importance of sequencing small fragments of paraffin-embedded samples, whose main application has been their storage for histopathological studies, when these samples could also prove to be a valuable input for the genomic surveillance necessary in host–pathogen studies for understanding the pathogenesis of different diseases. The use of sequencing tools in confirming diagnosis by DENV with greater specificity may be important in the surveillance and management of severe cases in endemic regions. In the present study, only 28% of the samples confirmed by sequencing of the envelope gene were evaluated due to the limiting nature of the inclusion in paraffin that can contribute to RNA degradation and therefore to the lower detection of RNA positive samples.

The lack of other serotypes could be because these serotypes were introduced on different dates than the cohort analyzed in this study; for example, the DENV-3 serotype was introduced in 2001, and the DENV-1 serotype was highly prevalent during the periods of 1998–1999, and 2007–2008, while the DENV-2 and DENV-4 serotypes showed the highest and lowest prevalence over time, respectively [50]. The high prevalence of DENV-2 can be attributed to the presence of highly infectious lineages, such as those from the Asian-American genotype. This genotype is highly associated with lethal outcomes after infection [46].

Our findings are similar to those reported in other infections associated with the DENV-2 serotype but also resemble those reported for autopsy studies linked to infections by other serotypes [35, 36], for example, in postmortem case studies of patients from Brazil and Vietnam infected with DENV-3, liver alterations such as hepatocellular necrosis, microvesicular steatosis, Kupffer cell hyperplasia, formation of councilman bodies and cellular infiltrate in the portal tract have been found. In these same patients, when evaluating the splenic tissue, interstitial edema and vascular and cellular congestion in the white pulp associated with reactive hyperplasia have been found [53]. In addition to the above, other case reports on deaths associated with DENV indicate atypical alterations in the kidney, lung, heart, and central nervous system in which hemorrhage, edema, and inflammatory infiltrate are observed, however still, in most cases, the serotype or genotype associated with viral infection is not reported [54].

In our study, it was impossible to make inferences between the level of damage and the viral genotype due to the limited number of samples used during the analysis. On the one hand, it is worth highlighting the implementation of specific primers for amplifying small fragments of the DENV-2 E gene by conventional PCR, which increased the probability of amplification and sequencing from fixed and embedded tissues. On the other hand, the lineages and genotypes obtained in the phylogenetic analysis agree with the viral strains that circulated during the epidemiological outbreak of 2010–2011 [29, 45].

## Supplementary Information

**Additional file 1: Table S1.** Summary of molecular findings and viral localization in tissue samples from DENV-2 fatal cases during the 2010 epidemic in Colombia.

**Additional file 2: Figure S1.** Agarose electrophoresis assays for detection of DENV-2 E gene fragment amplification products.

**Additional file 3: Table S2.** Main clinical signs reported in fatal cases of DENV-2 during the 2010 epidemic in Colombia.

## Data Availability

The sequences generated and analyzed during the current study are available in the National Center for Biotechnology Information (NCBI), [Accession numbers: OP435268; OP491462; OP491465; OP491529; OP491531; OP491530; OP491528; OP491597; Additional file 3: Table S2].

## Abbreviations

DENV: Dengue virus
DENV-1 to DENV-4: Dengue virus, serotypes 1 to 4
E Gen: Envolture gen
WHO: World Health Organization
DSS: Dengue shock syndrome
DHF: Dengue hemorrhagic fever
CIOMS: Council for International Organizations of Medical Sciences
RT‒PCR: Reverse transcription polymerase chain reaction
cDNA: Complementary DNA
