# Supplementary material for: Genotyping of dengue virus from infected tissue samples embedded in paraffin
Source: Virol J. 2023 May 25;20:100. doi: 10.1186/s12985-023-02072-5 (PMC10214625; doi:10.1186/s12985-023-02072-5)
Supplement: Supplementary file 1 — Additional file 1: Table S1. Summary of molecular findings and viral localization in tissue samples from DENV-2 fatal cases during the 2010 epidemic in Colombia. [file 12985_2023_2072_MOESM1_ESM.docx]

| **Pathology code** | **Organ** | **Inmmunohistochemistry (IHC)** | **Cell type positive for viral antigen** | **Negative Strand** | **ID Sample** | **Nucleic Acid Conc [ng/µL]** | **260/280** | **260/230** | **Genbank code** | **E sequence sizes pb** |
| --- | --- | --- | --- | --- | --- | --- | --- | --- | --- | --- |
| 59360 | Liver | Positive | Sinusoidal cells | 0 | S1 | 203.9 | 2.01 | 1.98 | OP491462 | 409 |
|  | Lung | Positive | Alveolar septum infiltrate cells | 1 | S2 | 98.7 | 2.01 | 2.05 |  |  |
| 59404 | Liver | Positive | Sinusoidal cells | 1 | S3 | 47.9 | 1.84 | 1.9 |  |  |
|  | spleen | Negative | Negative | 1 | S4 | 95 | 1.87 | 2.19 | OP491597 | 362 |
| 59439 | Liver | Positive | Sinusoidal cells | 1 | S5 | 230.9 | 1.94 | 2.22 |  |  |
|  | Lung | Negative | Alveolar septum infiltrate cells | 1 | S6 | 76.4 | 1.88 | 2.38 |  |  |
|  | Spleen | Positive | Negative | 1 | S7 | 19.6 | 2.08 | 3.44 | OP491530 | 399 |
|  | Heart | Negative | Negative | 1 | S8 | 25.2 | 1.68 | 1.15 |  |  |
| 59461 | Liver | Positive | Sinusoidal cells | 1 | S9 | 63.1 | 1.91 | 1.92 |  |  |
| 59479 | Liver | Positive | Hepatocytes, sinosoidal cells | 1 | S10-1 | 130.8 | 1.92 | 1.78 | OP491529 | 399 |
|  | Liver | Positive |  | 1 | S10-4 | 130.8 | 1.92 | 1.78 | OP491465 | 221 |
| 59484 | Liver | Positive | Sinusoidal cells | 1 | S11 | 193.9 | 1.85 | 1.97 |  |  |
|  | Liver | Positive | Sinusoidal cells | 1 | S12 | 165.4 | 1.83 | 1.93 |  |  |
| 59569 | Liver | Positive | Hepatocytes | 1 | S13 | 85.5 | 1.89 | 2.21 |  |  |
|  | Spleen | Positive | Connective septum | 1 | S14 | 49.2 | 1.87 | 2.32 | OP491528 | 409 |
|  | Lung | Negative | Negative | 1 | S15 | 241.3 | 1.85 | 2.06 |  |  |
|  | Heart | Positive | Connective tissue cells | 0 | S16 | 102.9 | 1.92 | 2.07 |  |  |
| 59616 | Liver | Positive | Sinusoidal cells | 1 | S17 | 281.8 | 2 | 2.14 | OP435268 | 369 |
|  | Liver | Positive | Sinusoidal cells | 1 | S18 | 69.7 | 1.9 | 2.09 |  |  |
| 59702 | Liver | Positive | Sinusoidal cells, connective tissue of Kiernan's space | 1 | S19 | 155 | 1.95 | 2.26 |  |  |
|  | Lung | Positive | Alveolar septum infiltrate cells | 1 | S20 | 76 | 1.88 | 2.3 |  |  |
| 59888 | Liver | Positive | Sinusoidal cells | 1 | S21 | 189.4 | 1.92 | 2.18 | OP491531 | 357 |
|  | Lung | Positive | Alveolar septum infiltrate cells | 1 | S22 | 155.6 | 1.97 | 2.21 |  |  |

**Note:** An absorbance method was used to assess RNA quality, taking into account the 260/230 and 260/280 ratios. A suitable extraction quality was determined to be values close to 2.0 and no lower than 1.8. A zero value on a negative strand indicates that it was not detected. The samples in which it was possible to obtain the amplification fragments are displayed in gray color.
