# Supplementary material for: Genotyping of dengue virus from infected tissue samples embedded in paraffin
Source: Virol J. 2023 May 25;20:100. doi: 10.1186/s12985-023-02072-5 (PMC10214625; doi:10.1186/s12985-023-02072-5)
Supplement: Supplementary file 2 — Additional file 2: Figure S1. Agarose electrophoresis assays for detection of DENV-2 E gene fragment amplification products. [file 12985_2023_2072_MOESM2_ESM.pdf]

Figure S1. Agarose electrophoresis assays amplifying DENV-2 E gene fragments. MP: Molecular weight marker, (+): positive control, (-): Negative control, numbers corresponding to samples in appendix: table 1.

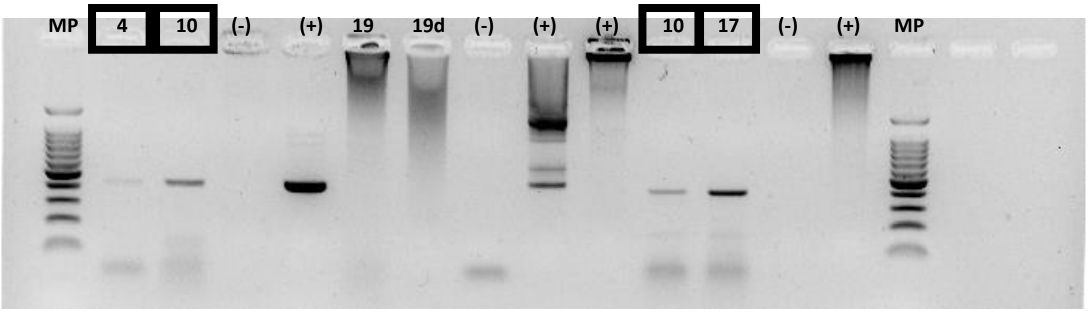

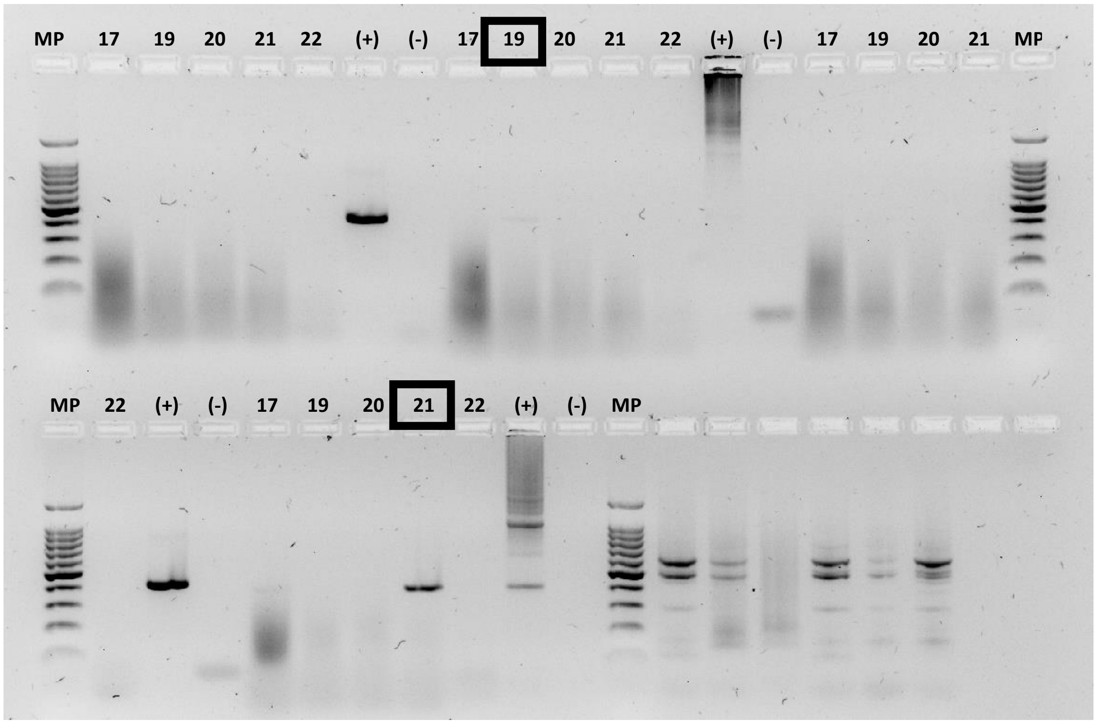

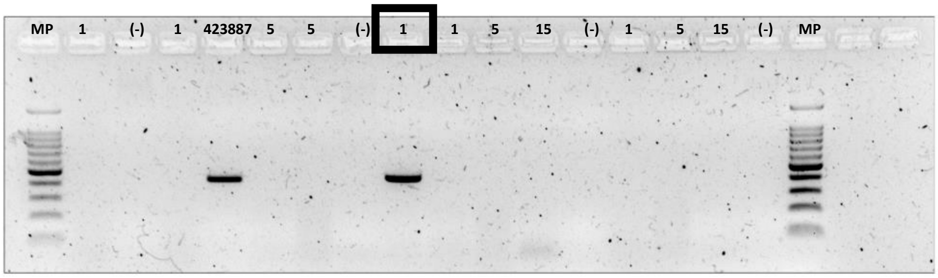

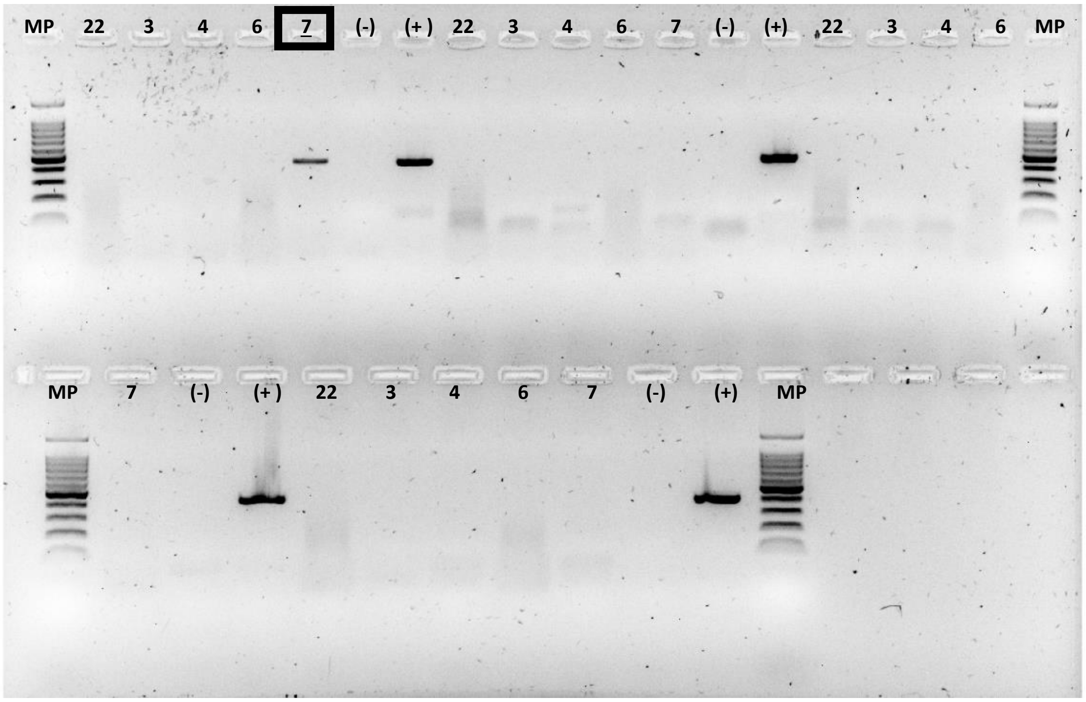
MP 22 3 4 6 7 (-) (+) 22 3 4 6 7 (-) (+) 22 3 4 6 MP
MP 7 (-) (+) 22 3 4 6 7 (-) (+) MP

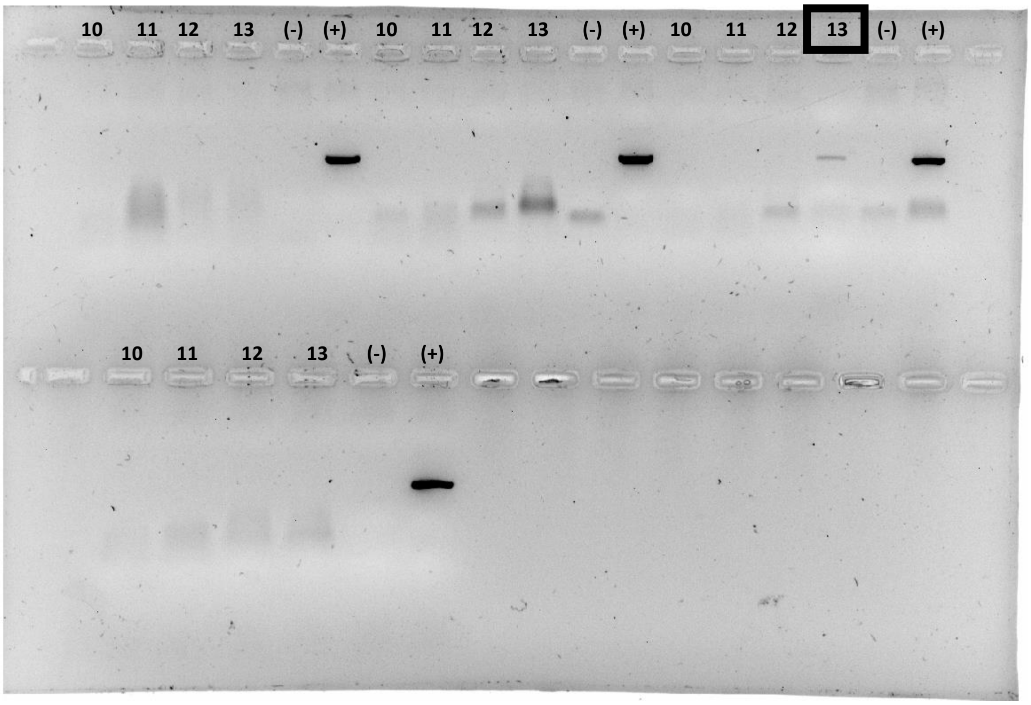
10 11 12 13 (-) (+) 10 11 12 13 (-) (+) 10 11 12 13 (-) (+)
10 11 12 13 (-) (+)
