# Supplementary material for: Genotyping of dengue virus from infected tissue samples embedded in paraffin
Source: Virol J. 2023 May 25;20:100. doi: 10.1186/s12985-023-02072-5 (PMC10214625; doi:10.1186/s12985-023-02072-5)
Supplement: Supplementary file 3 — Additional file 3: Table S2. Main clinical signs reported in fatal cases of DENV-2 during the 2010 epidemic in Colombia. [file 12985_2023_2072_MOESM3_ESM.docx]

| **Pathology Code /**  **ID Sample** | **59360**  S1-S2 | **59404**  S3-S4 | **59439**  S5-S8 | **59461**  S9 | **59479**  S10-1  S10-4 | **59484**  S11-S12 | **59569**  S13-S16 | **59616**  S17-S18 | **59702**  S19-S20 | **59888**  S21-S22 |
| --- | --- | --- | --- | --- | --- | --- | --- | --- | --- | --- |
| Age (Years) | 8 | 0,33 | 32 | 34 | 30 | 10 | 5 | 13 | 18 | 5 |
| Gender | M | F | M | M | F | F | M | F | F | F |
| Period between onset of symptoms and death (Days) | 4 | 4 | 5 | 14 | 5 | 4 | 7 | 10 | 7 | 3 |
| Myalgia | **ND** | 1 | 1 | 0 | 1 | 1 | 0 | 1 | 1 | 0 |
| Headache |  | 0 | 0 | 1 | 1 | 0 | 1 | 1 | 0 | 0 |
| Arthralgia |  | 0 | 1 | 0 | 0 | 0 | 0 | 1 | 0 | 0 |
| Abdominal pain |  | 1 | 0 | 1 | 1 | 0 | 1 | 1 | 0 | 1 |
| Encephalopathy |  | 0 | 0 | 1 | 0 | 0 | 0 | 0 | 0 | 0 |
| Hemorrhage |  | 1 | 0 | 1 | 1 | 0 | 1 | 1 | 0 | 0 |
| Jaundice |  | 0 | 0 | 0 | 0 | 0 | 0 | 0 | 0 | 0 |
| Hepatomegaly |  | 0 | 0 | 0 | 0 | 0 | 0 | 0 | 0 | 0 |
| AST/TGO |  | 1281,6 |  | 6820 | 1471 |  |  |  |  |  |
| ALT/TGP |  | 382,60 |  | 1842 | 405 |  |  |  |  |  |
| Platelets / μl | 34.000 | 49.000 | 70.000 | 12.000 | 3.000 | 47.000 | 33.000 | 53.000 | 25.000 |  |
| Hematocrit % | 26,20 | 22,2 |  | 47,80 | 24,50 |  | 40,80 | 31,10 |  |  |

Reference values: **Platelets** all ages (150.000 – 450.000/μl); **Hematocrit**, 3-6 months (29-41%), 6-12 years (35-45%), >18 years female (33-51%), >18 years male (37-53%).

- Nathan DG, and Oski, FA (1981) Hematology of Infancy and Childhood, ed 2, WB Saunders, pp 1552-74.

- Dallman PR (1977) Pediatrics, 16th edition, Appleton-Century-Crofts, p 1111

- Pediatric Normal Ranges (1995) Children’s Hospital of Buffalo, Coulter VIEWPOINT, No. 17, p 8

- Pediatric Normal Range Study (1995) Children’s Hospitals, Minneapolis and St. Paul
